# Editorial: Computational Behavioral Modeling for Big User Data

**DOI:** 10.3389/fdata.2022.893216

**Published:** 2022-04-13

**Authors:** Meng Jiang, Chuxu Zhang, Xiangliang Zhang, Neil Shah

**Affiliations:** ^1^Department of Computer Science and Engineering, University of Notre Dame, Notre Dame, IN, United States; ^2^Department of Computer Science, Brandeis University, Waltham, MA, United States; ^3^Snap Inc., Santa Monica, CA, United States

**Keywords:** user modeling, behavior modeling, representation learning, group behavior, data mining

The research within this Topic aims at designing, developing, evaluating, and evaluating computational models for tasks such as pattern analysis, prediction, recommendation, and anomaly detection, on large scale user datasets. With the massive amounts of user data currently available and being collected, obtaining access to data is seldom the concern. Information is being produced and stored at an unprecedented rate, and increasingly, much of the big data being collected is about human behavior. User behavior is captured in the information that we provide from using web search engines, e-commercial platforms, social network services, or online education. Sifting through this data and deriving insights on human behavior enables the platforms to make more effective decisions and provide better service. However, traditional behavior modeling mainly relies on qualitative methods from behavioral science and social science perspectives.

This Research Topic reports four innovative solutions to problems of user behavior data scale in a wide range of applications such as recommender systems and suspicious behavior detection. It covers data science and statistical approaches to knowledge discovery and modeling, decision support and prediction, including machine learning and AI, on user behavior data. Key findings of these solutions are as follows: (1) Representation learning methods such as matrix factorization and graph neural networks are effective for understanding user generated content and behaviors. (2) Group-level behaviors need careful investigation toward downstream tasks such as detecting suspicious group behaviors and identifying polarization. Next paragraphs present four contributing articles of this Research Topic:

The first article titled “*Unified Representation of Twitter and Online News using Graph and Entities*” is contributed by Syed et al. When online news services employ strategies for personalizing and recommending articles to their users based on their interests, this work aims to improve consumer engagement and satisfaction. It presents a novel model that builds a generalized graph of user generated contents including news articles and tweets. The model delivers promising results on downstream tasks such as identifying sentiment, trending topics, and misinformation.

The second article titled “*Detecting Group Anomalies in Tera-Scale Multi-Aspect Data via Dense-Subtensor Mining*” is contributed by Shin et al. It aims to detect fraudulent lockstep behavior in large-scale multi-aspect data which can be represented as tensors. The goal is to perform efficient detection when data are too large to fit in memory or even on a disk. Existing methods have low accuracy, or they assume that tensors are small enough to fit in main memory, which is unrealistic in many real-world applications such as social media and web. This work proposes a disk-based dense-subtensor detection method, which also can run in a distributed manner across multiple machines. It is memory efficient, fast, provably accurate, and effective.

The third article titled “*Computational Modeling of Hierarchically Polarized Groups by Structured Matrix Factorization*” is contributed by Sun et al. The objective is modeling hierarchically polarized groups on social media. This work introduces an enhanced unsupervised non-negative matrix factorization algorithm for computational modeling of hierarchically polarized groups. This algorithm aims to detect points of agreement and disagreement between groups and divide them hierarchically to represent nested patterns of agreement and disagreement. It is enhanced with a language model, and with a proof of orthogonality of factorized components. Experiments show that it outperforms state-of-the-art approaches for polarization detection and stance separation. An ablation study further illustrates the value of individual components, including new enhancements.

The fourth article titled “*Recipe Recommendation With Hierarchical Graph Attention Network*” is contributed by Tian et al. Recipe recommendation systems play an important role in helping people find recipes that of their interest and fit their eating habit. It proposes a novel hierarchical graph attention network for recipe recommendation. This model can capture user history behavior, recipe content, and relational information through several neural network modules, including type-specific transformation, node-level attention, and relation-level attention. This work further introduces a ranking-based objective function to optimize the model. Experiments demonstrate that the new model outperforms numerous baseline methods.

## Author Contributions

MJ, CZ, XZ, and NS organized the Research Topics, invited contributions, and reached out to quality reviewers to review the submissions. All authors contributed to the article and approved the submitted version.

## Conflict of Interest

NS was employed by Snap Inc. The remaining authors declare that the research was conducted in the absence of any commercial or financial relationships that could be construed as a potential conflict of interest.

## Publisher's Note

All claims expressed in this article are solely those of the authors and do not necessarily represent those of their affiliated organizations, or those of the publisher, the editors and the reviewers. Any product that may be evaluated in this article, or claim that may be made by its manufacturer, is not guaranteed or endorsed by the publisher.

